# Patterns of RNA Editing in Newcastle Disease Virus Infections

**DOI:** 10.3390/v12111249

**Published:** 2020-11-02

**Authors:** Archana Jadhav, Lele Zhao, Alice Ledda, Weiwei Liu, Chan Ding, Venugopal Nair, Luca Ferretti

**Affiliations:** 1 Viral Oncogenesis group, The Pirbright Institute, Pirbright, Woking, Surrey GU24 0NF, UK; archu15488@gmail.com (A.J.); venugopal.nair@pirbright.ac.uk (V.N.); 2 Big Data Institute, Li Ka Shing Centre for Health Information and Discovery, Nuffield Department of Medicine, University of Oxford, Oxford OX3 7LF, UK; lele.zhao@bdi.ox.ac.uk; 3 MRC Centre for Global Infectious Disease Analysis, Department of Infectious Disease Epidemiology, School of Public Health, Imperial College London, London W2 1NY, UK; a.ledda@imperial.ac.uk; 4 Department of Avian Infectious Diseases, Shanghai Veterinary Research Institute, Chinese Academy of Agricultural Science, Shanghai 200241, China; liuweiwei@shvri.ac.cn (W.L.); shoveldeen@shvri.ac.cn (C.D.); 5 UK-China Centre of Excellence on Avian Disease Research, Pirbright, Guildford, Surrey GU24 0NF, UK

**Keywords:** polymerase stuttering, pseudo-templated transcription, deep sequencing

## Abstract

The expression of accessory non-structural proteins V and W in Newcastle disease virus (NDV) infections depends on RNA editing. These proteins are derived from frameshifts of the sequence coding for the P protein via co-transcriptional insertion of one or two guanines in the mRNA. However, a larger number of guanines can be inserted with lower frequencies. We analysed data from deep RNA sequencing of samples from in vitro and in vivo NDV infections to uncover the patterns of mRNA editing in NDV. The distribution of insertions is well described by a simple Markov model of polymerase stuttering, providing strong quantitative confirmation of the molecular process hypothesised by Kolakofsky and collaborators three decades ago. Our results suggest that the probability that the NDV polymerase would stutter is about 0.45 initially, and 0.3 for further subsequent insertions. The latter probability is approximately independent of the number of previous insertions, the host cell, and viral strain. However, in LaSota infections, we also observe deviations from the predicted V/W ratio of about 3:1 according to this model, which could be attributed to deviations from this stuttering model or to further mechanisms downregulating the abundance of W protein.

## 1. Introduction

Newcastle disease (ND) is a well-known, economically important, and prevalent poultry disease worldwide. The causative agent of ND is Newcastle disease virus (NDV), which belongs to the *Orthoavulavirus* genus of *Avulavirinae* subfamily in the *Paramyxoviridae* family [1]. NDV is an enveloped virus and, like any other paramyxovirus, has an approximately 15 kb long non-segmented, single-stranded, negative sense RNA genome. NDV genome encodes six essential genes expressing main structural proteins in the order of 3′-NP-P-M-F-HN-L-5′ [2], which are nucleocapsid protein (NP), phosphoprotein (P), matrix protein (M), fusion protein (F), haemagglutinin-neuraminidase protein (HN), and large RNA-dependent RNA polymerase (L) [3]. Each NDV gene contains conserved transcriptional regulatory sequences known as gene start (GS) 3′-UGCCCAUCU/CU-5′ and gene end (GE) 3′-AAUU/CC/UU_5-6_-5′, respectively. In between two genes, there are intergenic sequences (IGSs), which span from 1 to 47 nucleotides.

NDV HN, F, and M proteins are components of the viral envelope. NDV-HN protein interacts efficiently with α-2,3 and α-2,6 N-linked sialic acid conjugates [4]. The HN protein and cell receptor tethering activate the F protein, which facilitates fusion activity for viral entry and egress [5]. Meanwhile, M protein is responsible for the translocation of viral components and virion assembly at the host cell membrane [6]. The NP is an RNA binding protein where monomers of NP proteins bind to full length genomic (−ve) and antigenomic (+ve) RNAs and form encapsidated and helical structured biologically active template for RNA transcription and replication in the host cell cytoplasm [7]. NDV P protein is an important component of viral RNA polymerase enzyme complex and essential for viral RNA synthesis [8]. NDV also expresses two non-structural accessory proteins by mRNA editing of the P gene at the preserved editing site (3′-UUUUUCC-5′). P gene uses non-template guanine residues (G), viz. +G (V) and +GG (W), during co-transcription modification, resulting in a shift of the respective open reading frame (ORF), where P, V, and W proteins share the amino-terminal, but have a distinctive carboxy-terminal. The relative approximate proportion of NDV proteins P/V/W reported for P is 60 to 70%, for V is 25 to 35%, and for W is 2 to 8.5% in NDV-infected cells [9]. The insertion of more than two G residues leading to the supplementary amino acid insertion is rare, but possible in chicken cells [9,10].

The V protein plays roles in the NDV virulence as it antagonises IFN responses. The carboxy-terminal of V protein inhibits type-I IFNs (IFN-α/β) signalling by targeting signal transducer and activator transcription factor 1 (STAT-1). The carboxy-terminal domain of V protein interacts with melanoma differentiation-associated protein 5 (MDA5) to impede IFN-β response [11,12]; however, the function of the W protein remains unclear. NDV L protein is a central subunit of RNA polymerase complex, which controls enzymatic activities required for genomic RNA transcription into functional viral mRNA, nucleotide polymerisation, mRNA post-transcriptional modification by 5′ methyl cap and 3′ poly-A tail, as well as replication of biologically active genomic and antigenomic RNA [6]. In NDV-infected cells, P and L proteins start viral RNA synthesis at the 3’ end of genomic RNA to form leader RNA by the start and stop mechanism at each gene junction directed by GS and GE sequences [6]. The NDV genome, like other paramyxoviruses, contains multiple hexamers of nucleotides and follows ‘the rule of six’ to maintain efficient replication and transcription as the RNA is encapsidated in a helical structure by the NP protein, with each NP protein monomer spanning six nucleotides of the genome [13,14].

A unique feature of RNA editing in the P gene and the use of an alternate frame of NDV and other paramyxoviruses is known to increase the genome coding capacity of the virus efficiently [15]. The co-transcriptional RNA editing of P gene in paramyxoviruses is thought to be based on the stuttering mechanism of RNA polymerase enzyme in the ORF region in a similar fashion, where the polyadenylated tail is added to each transcript at the 3′ end of mRNA [16,17]. A model for the stuttering mechanism has been proposed by Kolakofsky and collaborators in 1990 [16,18]. The model suggested that, during transcription, the nascent chain of mRNA is weakly paired with the genomic template. When polymerase transcription complex halts at the editing site, the nascent mRNA would disassociate and realign with the template RNA, thus introducing G insertions [16].

The stuttering mechanism of co-transcriptional mRNA editing suggests that a larger number of guanines can be inserted with lower frequencies, and in fact, such insertions have been observed and are not uncommon among paramyxoviruses [19]. While these longer insertions are rare in NDV and thus unlikely to play any significant role, they are generated by the same stuttering mechanism. Hence, a more accurate study of the distribution of such insertions could provide a quantitative test of this stuttering model.

In this paper, we analyse data from deep mRNA sequencing to uncover the patterns of mRNA editing in NDV. We consider different strains from in vitro and in vivo infections: LaSota and Herts/33 infections in cultures of CEF cells, and LaSota experimental infections of Leghorn and Fayoumi chicken. We build a simple Markov model of polymerase stuttering to describe the distribution of guanine insertions and the regulation of the relative abundance of W and V, reproducing the basic features of polymerase stuttering proposed by Kolakofsky [16]. We apply this model to deep sequencing data from all samples, in order to provide a quantitative understanding of the stuttering process. We find a very good agreement, but also observe some deviations from this model, possibly related to further mechanisms affecting the regulation of the relative abundance of W and V.

## 2. Materials and Methods

### 2.1. Datasets

We analysed samples from in vitro NDV infections in chicken embryo fibroblast (CEF) cells using LaSota and Herts/33 strains [20] and in vivo NDV infections from Leghorn and Fayomi chicken lines using LaSota strain [21,22,23]. These chicken lines differ in their phenotype, with Leghorn being more susceptible and Fayoumi more resistant to NDV infections. We report results on only six in vivo samples (three from Leghorn and three from Fayoumi), as they were the only ones with a reasonably high depth of viral reads at the editing site. The details of the samples are summarised in Table 1 and Table S1.

Cultured CEF cells were infected with LaSota or Herts/33 at a MOI of 1 and incubated at 37 °C with 5% CO_2_. They were later cultured in 2% FBS containing DMEM and harvested before 12 h post infection. For in vivo studies, 21-day-old Fayoumi and Leghorn chickens were infected with 200 μL of 10^7^ embryos infectious dose (EID) of 50% through intranasal and ocular routes, then the trachea was harvested 2 days post infection.

RNA sequencing was performed on Illumina HiSeq2500 platforms (Illumina Inc., San Diego, CA, USA), with paired-end sequencing of 125 bp reads for the in vitro experiments and single-end sequencing of 100 bp reads for the in vivo ones. More details on the experimental design, preparation, and sequencing of the datasets analysed here can be found in the original publications.

It has been shown that sequencing of mRNA isolated by poly(A) selection can lead to biases in quantification of expression, because of the contribution of genomic and antigenomic RNA [24]. However, the amount of antigenomic RNA should be negligible compared with genomic RNA and mRNA, as it is used as a template for viral replication [24]. Given the strong 3′ to 5′ gradient in the expression of NDV genes, we can quantify an upper bound on the contribution of genomic RNA by comparing the expression of mRNAs coding for L (the least expressed gene, which is thus an upper bound on the amount of genomic RNA) and P. The resulting upper bound on genomic RNA contribution is 2 to 5% for the samples from in vivo infection but reaches 80 to 100% for the samples from infected CEFs (Table S2). Hence, the actual fraction of genomic RNA (which is not edited) is negligible in the former samples, but unknown in the latter samples.

### 2.2. Bioinformatic Analysis

As viral reference, we used the LaSota reference sequence with GenBank accession JF950510 (complete genome of NDV LaSota strain-15186 bp cRNA linear) and Herts/33 sequence AY741404 (complete genome of NDV Herts/33 strain-15186 bp RNA) for the data from in vitro experiments in [20], and LaSota sequence AF077761 (complete genome of NDV LaSota strain-15186 bp RNA) for the in vivo data from [21,22,23]. Reads were aligned to the combined transcriptome of NDV and Gallus gallus (genome build GRCg6a, gene build 2018-03) using the RNA-pipeline from the GEM aligner [25] with default parameters. We realigned all indels across all samples using LeftAlignIndels from GATK 4.0.1.2 [26] in order to assign all insertions near the RNA editing site to a single location in the genome. Reads were filtered for mapping and base quality >30 using SAMtools [27]. Variants were called using SiNPle v1.0 [28] with default parameters.

We verified manually that SAMtools mpileup assigned all insertions to positions 2284 with respect to the sequence of AF077761, and to position 2287 with respect to JF950510 and AY741404, corresponding to the base before the start and end position of the guanine homopolymeric stretch that is expanded by RNA editing. Hence, we considered all homopolymer insertions of guanines (+G) of any multiplicity at the start position for AF077761 and at the end position for JF950510 and AY741404. Finally, we considered only the samples that were covered by at least 100 reads at these positions.

To make sure that the insertions were NDV-specific and not more general artefacts of sample preparation and sequencing (e.g., stuttering of the polymerase in vivo/in vitro during the experiments or from reverse transcriptase and amplification), we performed a semi-automatic search for single-base insertions at frequencies >1% supported by at least two NDV or chicken reads, selected the ones that also included double insertions in the same position, then screened manually the resulting positions looking for insertions of homopolymeric sequences of variable length. We found such positions once every 3 to 5 kb of sequence, in both NDV and in human transcriptome; these insertions were often shared among replicates from the same experiment, but differed among experiments, even if performed by the same group. These artefacts are thus likely to depend both on the experimental protocol, on the host and on NDV strain, and possibly on the specific run of extraction and preparation; it is then unlikely that such artifacts would precisely affect the position in the NDV genome corresponding to the RNA editing process by the NDV polymerase.

### 2.3. Markov Model of Polymerase Stuttering

**Figure 1:**
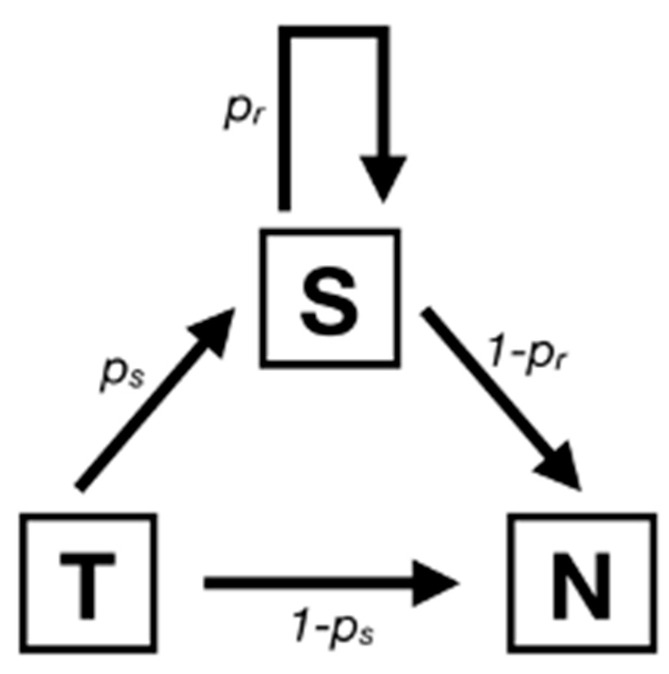
Markov model of polymerase stuttering. In this model, three states are possible: Transcribe, Stutter, Next. The two rates in the figure can be inferred from deep sequencing data.

To describe the process of polymerase stuttering, we implemented a Markov model (Figure 1) with three possible states: transcribe (T), next (N), and stutter (S). In normal conditions, the polymerase would proceed from the state T where it just transcribed a base to the state N, where it would transcribe the next base. In our model, this transition happens with probability $1-p_{s}$. The system goes in a stuttering state (S) with probability $p_{s}$. This means that the same guanine base is transcribed once more, resulting in a single insertion. The polymerase can then stutter again with a different probability p_r_ or the polymerase might move to the next base, ending up in the absorbing state N (corresponding to the transcription of the rest of the sequences) with probability 1 − p_r_. After the first time, the polymerase can stutter any number of times, each time with probability p_r_.

The probability of the polymerase not stuttering, resulting in no insertion at all, is

(1) $$p\left(l=0\right)=1-p_{s}$$

while the probability of a stutter of length *l* > 0 (i.e., insertion of a homopolymeric stretch of *l* guanines) is

(2) $$p\left(l\right)=p_{s}p_{r}^{l-1}\left(1-p_{r}\right)$$

A fraction γ of genomic RNA would change the observed distribution of the length of the insertion as

(3) $$p{\left(l\right)}_{observed}=\left(1-\gamma \right)p\left(l\right)+\delta_{l,0}\gamma$$

where δ_*l*,0_ = 1 for *l* = 0 and 0 otherwise. This would also change the inferred fraction of V and W, by reducing both of them by a factor 1 − γ; however, their ratio is unchanged.

We consider the logarithm (in base 10) of p(l). Our model predicts a linear dependence on l for this quantity, except for l = 0. The slope of the linear part is $log_{10}\left(p_{r}\right)$, while its intercept is $log_{10}(p_{s}\left(1-p_{r}\right)/p_{r})$, provided that the contribution from genomic RNA is negligible.

## 3. Results

### 3.1. RNA Editing and P/V/W Frequencies

The relative frequencies of P, V, and W mRNA that can be inferred from the frequencies of different insertions among the reads are reported in Table 2.

The apparent fraction of P is very different among different samples (Table S3); however, because of the unknown contribution of genomic RNA to the reads from CEF cells, the quantification of P could be overestimated.

**Figure 2:**
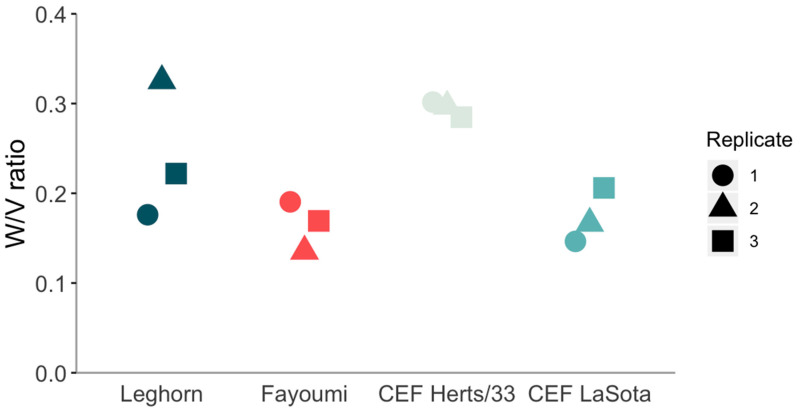
*W*/*V* ratio for different datasets.

The *W*/*V* ratio is not affected by the unknown contribution from genomic RNA. The values of *W*/*V* are in the range of 0.14 to 0.33, with a mean of 0.22, median of 0.2, and SD of 0.07 (Figure 2). Values from in vivo infections are not dissimilar to the ones from in vitro infection of the same LaSota strain but differ significantly from the ones of the virulent Herts/33 strain (Table 3).

### 3.2. Pattern of Polymerase Stuttering

**Figure 3:**
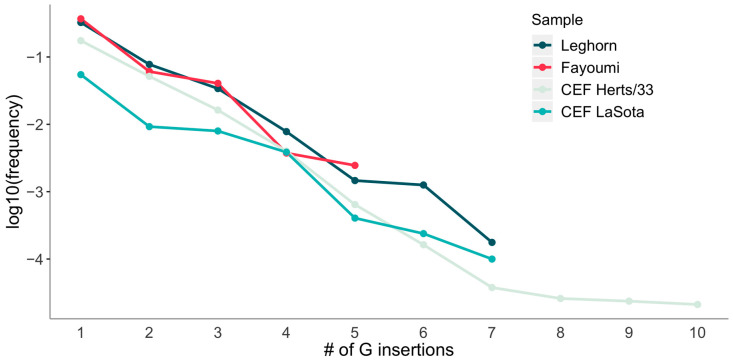
Log-plot of mRNA fraction with a given length of G insertions, averaged among all samples from the same experiment.

To understand what controls the P/V/W ratio, we explore the patterns of insertions of more than two guanines. Interestingly enough, for all four experiments, we observe a clear geometric decay (i.e., a linear decay in log-scale, see Figure 3).

**Figure 4:**
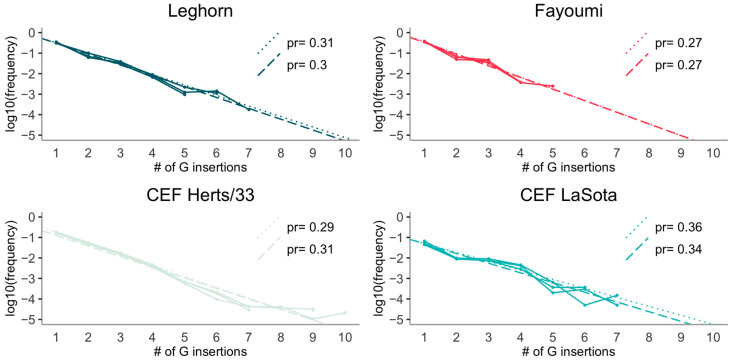
Log-plots of mRNA fraction as a function of insertion length, presented for each sample separately. Straight lines show the linear regressions for each experiment (the dotted line corresponds to the “Poisson-weighted” regression).

Our simple Markov model of stuttering predicts a geometric decay as well. We fit both an unweighted linear model (assuming that the measurement variance scales as the square of the frequency) and a linear model weighted according to an uncertainty on frequencies due to Poisson noise (i.e., assuming that the measurement variance between samples scales as the read count). A linear decay in log-scale fits our data very well, no matter the assumptions about the uncertainties in the frequencies (Figure 4).

It is remarkable that, fitting a linear model for each experiment (Figure 4), all coefficients provide an estimate for $p_{r}≃0.33$ (i.e., a slope of about −0.5 in the log10 plot). The same is true for a joint linear fit of all experiments, allowing for different slopes. All differences in slope (and thus in $p_{r}$) between experiments are non-significant (Table 4).

It is even more remarkable that, for the in vivo samples, which contain a negligible fraction of genomic RNA, the weighted fit (Table 4) predicts a value for $p_{s}≃0.45$, i.e., larger, but not so far from $p_{r}$ (Figure S1).

These conclusions do not change if we fit an unweighted linear model (Figure 4, Table S4).

### 3.3. Further Suppression of W mRNA Expression

**Figure 5:**
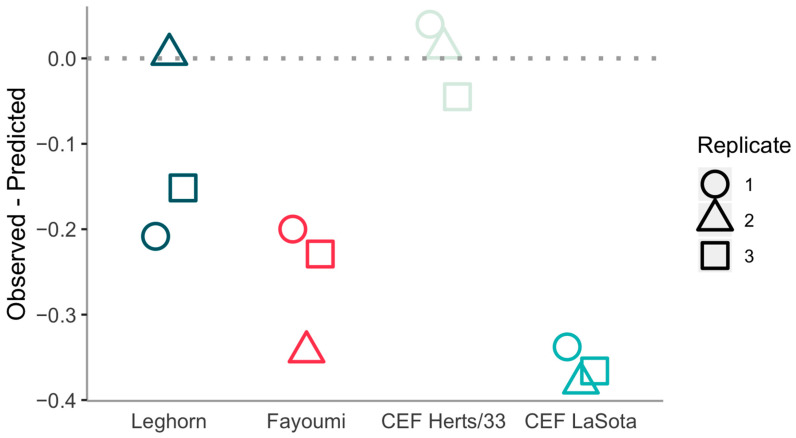
Difference between the actual log10-fraction of reads with +GG insertions and the predicted one based on our model fitted on all but +GG insertions.

We also note a systematic deviation from the model. While double insertions +GG closely follow the trend described by the model for Herts/33 infections in CEF cells, the scenario is different for all LaSota samples. In all LaSota infections but one, +GG insertions appear to be underrepresented with respect to the predictions of the model. We reanalysed the stuttering profile ignoring +GG insertions and compare their fraction with the predicted one in Figure 5.

This further suppression of +GG insertions significantly reduced the fraction of W mRNA in all LaSota samples (Figure 2), as +GG insertions represent the dominant source of W mRNA. As already observed, the difference is statistically significant (Table 3). The deviation is clearly attributable to double insertions, as longer insertions follow the model reasonably well without any clear trend in their deviations from it.

## 4. Discussion

RNA editing in paramyxoviruses is one of the most interesting examples of a functional role for pseudo-templated transcription and polymerase stuttering [29]. The evidence presented by Kolakofsky and collaborators for polymerase stuttering in NDV as the origin of V and W proteins [17,19] was very convincing. Three decades after their proposal, our work has provided strong quantitative support for their model, also revealing some unexpected relations between its parameters.

We find a value of $p_{r}≃0.3$ for the probability of stuttering, which appears to be remarkably universal among all infections studied here. This would hint at a universal value for the *W*/*V* ratio, if such a ratio would be controlled purely by polymerase stuttering. However, we also observe evidence of a difference between Herts/33 and LaSota in the *W*/*V* ratio, with a lower ratio than expected for both in vivo and in vitro LaSota-infected samples, compared with the good fit of our stuttering model. This lower *W*/*V* ratio corresponds to a lower amount of mRNA with +GG insertions. Hence, the regulation of W expression is likely to be more complicated than conjectured and controlled by further mechanisms beyond simple polymerase stuttering. Based on our finding, the simplest explanations would be either a preference for +GG insertions, similarly to what happens at the first round of insertions in other paramyxoviruses [19], but only during the second stuttering event, or a hypothetical mechanism suppressing specifically the expression of mRNA with double guanine insertions.

Inference of the fraction of P mRNA from CEF cells is unfortunately unreliable because of the contribution from genomic RNA [24]; consequently, the inference of $p_{s}$ is unreliable from in vitro samples. It is, however, tempting to speculate, on the basis of evidence from the in vivo data only, that $p_{s}≃0.45$, i.e., that after the initial insertion of a guanine, further stuttering by the polymerase would be slightly inhibited. This would be consistent with suggestions that, after the first round of insertions, there could be either an inhibition of the pause needed to stutter [29], or a displacement of the strand being formed [18]. Such a scenario is likely to also occur for measles virus, where evidence could hint at $p_{s}≃0.3,\;p_{r}≃0.1$ [30]. If this would be the case, the two parameters $p_{r},\;p_{s}$ would then control both the V/P ratio and the *W*/*V* ratio, but for the extra regulation discussed above. Interestingly, the opposite relation between rates ($p_{r}>p_{s}$) was hypothesised to explain the results of some upstream sequence modifications [31]. It should be remarked that there are large uncertainties on the value of $p_{s}$, thus the two parameters could also have a similar value.

Our results show how RNA processes such as mRNA editing can be detected and quantified from deep sequencing data. They also illustrate the richness of information that can be extracted from massive sequencing datasets: from the regulation of the host transcriptome [20,21,22,23], to the genetic diversity of the viral population (A. Jadhav et al., in preparation), and to the RNA modifications presented here. More generally, this work illustrates the power of creative applications of modern sequencing technologies in shedding light on aspects of the molecular biology of RNA viruses.

## Figures and Tables

**Table 1:** Summary of samples used from in vivo and in vitro experiments. NDV, Newcastle disease virus; CEF, chicken embryo fibroblast.

Melissa Deist & Lamont Lab Group [21,22,23]					Prof Chan Ding’s Lab [20]	
Samples	Chicken Line	Sex	Phenotype	Chicken Age	Samples	Embryo Age
Leghorn rep 1	Leghorn	Female	Susceptible	21	LaSota rep 1	CEF cells isolated from 10-day-old SPF chicken embryos
Leghorn rep 2	Leghorn	Female	Susceptible	21	LaSota rep 2	
Leghorn rep 3	Leghorn	Male	Susceptible	21	LaSota rep 3	
Fayoumi rep 1	Fayoumi	Female	Resistant	21	Herts/33 rep 1	
Fayoumi rep 2	Fayoumi	Female	Resistant	21	Herts/33 rep 2	
Fayoumi rep 3	Fayoumi	Male	Resistant	21	Herts/33 rep 3	
Virus dose	200 microliters 10^7^ embryo infectious dose of 50%				Virus dose	MOI = 1
Experiment type	in vivo				Experiment type	in vitro
Organ harvested	Trachea				Organ used for primary cells	Chicken embryo
Cell type	Epithelial cell				Cell type	Fibroblast cell
Sample type	RNA				Sample Type	RNA
Time of tissue harvest	2 days post infection				Time of cell harvest	12 h post infection
NDV strain	LaSota (non-pathogenic)				NDV strain	LaSota (non-pathogenic) & Herts/33 (highly pathogenic)

**Table 2:** Frequencies and counts of reads that can be attributed to P, V, and W mRNA.

Samples	P	V	W	P mRNA Count	V mRNA Count	W mRNA Count
Leghorn 1	0.5881057	0.3502203	0.0616740	534	318	56
Leghorn 2	0.5895536	0.3096877	0.1007588	3341	1755	571
Leghorn 3	0.6131687	0.3165559	0.0702754	1937	1000	222
Fayoumi 1	0.5700246	0.3611794	0.0687961	464	294	56
Fayoumi 2	0.5855513	0.3650190	0.0494297	154	96	13
Fayoumi 3	0.5555556	0.3801170	0.0643275	95	65	11
CEF Herts/33 1	0.7581556	0.1857977	0.0560466	71,789	17,593	5307
CEF Herts/33 2	0.7703368	0.1770732	0.0525900	79,934	18,374	5457
CEF Herts/33 3	0.7920412	0.1618567	0.0461021	75,335	15,395	4385
CEF LaSota 1	0.9236920	0.0665634	0.0097446	18,484	1332	195
CEF LaSota 2	0.9380527	0.0531048	0.0088425	18,883	1069	178
CEF LaSota 3	0.9465327	0.0443343	0.0091330	20,624	966	199

**Table 3:** *p*-values of Student’s t-test for differences in ratio of *W*/*V* mRNA.

*p*-Value	Leghorn	Fayoumi	CEF Herts/33	CEF LaSota
Leghorn	1.0000000	0.1803779	0.2960698	0.2243687
Fayoumi	0.1803779	1.0000000	0.0015257	0.7551445
CEF Herts/33	0.2960698	0.0015257	1.0000000	0.0026214
CEF LaSota	0.2243687	0.7551445	0.0026214	1.0000000

**Table 4:** Combined linear model for log10 (mRNA fraction) as a function of experiments and length of insertions, weighted by read count (corresponding to inverse variance due to Poisson noise in read sampling).

Coefficient	Estimate	Std. Error	*t* Value	Pr (>|t|)
(intercept)	0.0091800	0.0549286	0.1671257	0.8677600
*l* (slope)	−0.5130190	0.0339636	−15.1049786	0.0000000
Fayoumi	0.1106835	0.1650741	0.6705082	0.5047720
CEF-LaSota	−0.2277560	0.0566429	−4.0209119	0.0001460
CEF-Herts/33	−0.8588694	0.0722928	−11.8804256	0.0000000
*l*:Fayoumi	−0.0589679	0.1098642	−0.5367340	0.5931778
*l*:CEF-LaSota	−0.0224315	0.0350717	−0.6395889	0.5245578
*l*:CEF-Herts/33	0.0737953	0.0428570	1.7218949	0.0895690

## Supplementary Materials

The following are available online at https://www.mdpi.com/1999-4915/12/11/1249/s1, Table S1: information about samples, Table S2: L/P coverage ratio as upper bound on the contribution of genomic RNA to the reads, for each sample in our datasets, Table S3: p-values of Student’s t-test for differences in fraction of P mRNA, Table S4: combined linear model for log10(mRNA fraction) as a function of experiments and length of insertion, Figure S1: distributions of inferred values of $p_{r},\;p_{s}$ from the weighted linear model presented in the Main Text, assuming independent Gaussian uncertainties for each term of the regression.
