# Supplementary material for: Patterns of RNA Editing in Newcastle Disease Virus Infections
Source: Viruses. 2020 Nov 2;12(11):1249. doi: 10.3390/v12111249 (PMC7693698; doi:10.3390/v12111249)
Supplement: Supplementary file 1 [file viruses-12-01249-s001.pdf]

Supplementary Figures and Tables

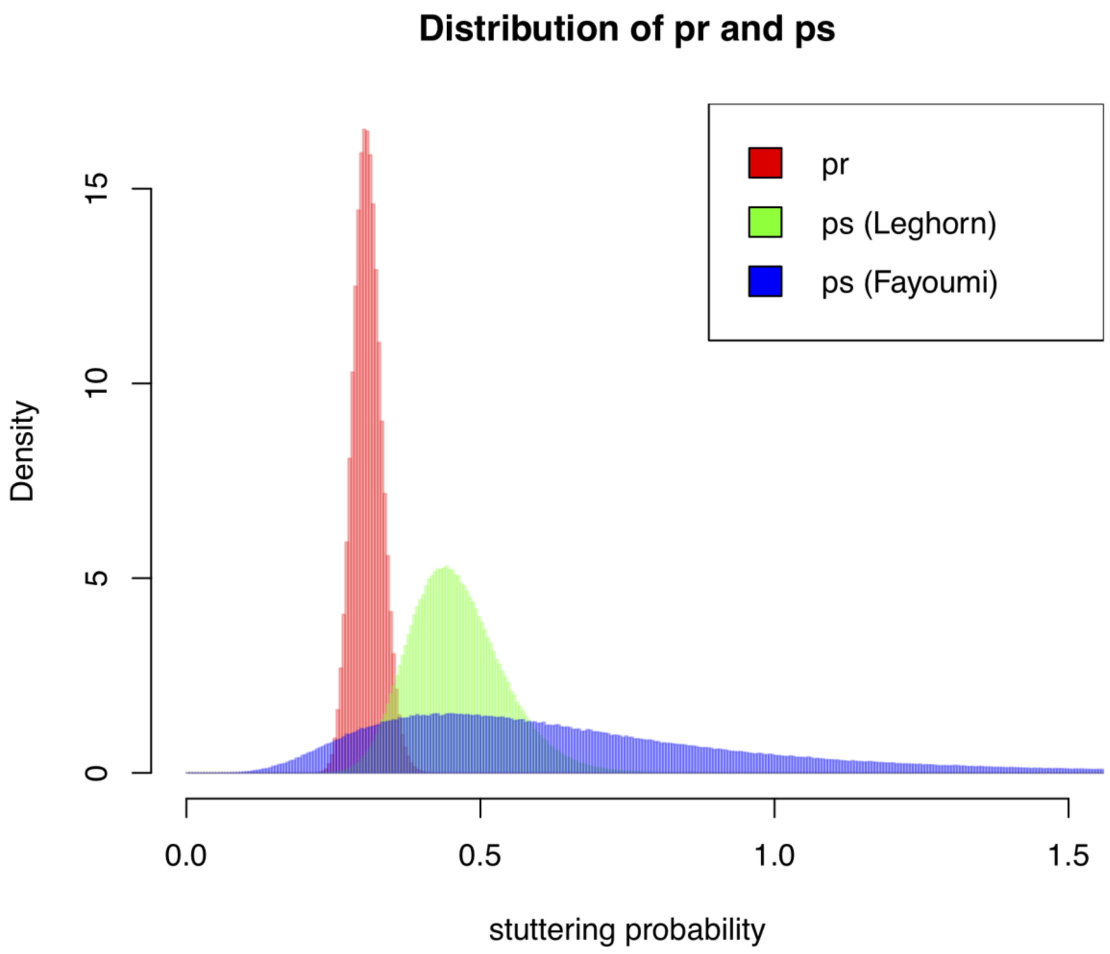

Supplementary Figure 1: distributions of inferred values of p_r, p_s from the weighted linear model presented in the Main Text, assuming independent Gaussian uncertainties for each term of the regression.

Supplementary Table 1: information about samples.

Study type	Samples	Sample ENA-Run	Chicken Line	Sex	Age in days	Organ	Cell Type	Virus	Viral Dose	Time points	Reference
In vivo	Sample 28	ERR1817162	Leghorn	Female	23	Trachea	Epithelial cell	LaSota	200 microliters 10^7 embryo infectious dose of 50%	2 dpi	[23]
In vivo	Sample 32	ERR1817163	Leghorn	Female	23	Trachea	Epithelial cell	LaSota	201 microliters 10^7 embryo infectious dose of 50%	2 dpi	[23]
In vivo	Sample 30	ERR1817164	Leghorn	Male	23	Trachea	Epithelial cell	LaSota	202 microliters 10^7 embryo infectious dose of 50%	2 dpi	[23]
In vivo	Sample 42	ERR1817166	Fayoumi	Female	23	Trachea	Epithelial cell	LaSota	204 microliters 10^7 embryo infectious dose of 50%	2 dpi	[23]
In vivo	Sample 43	ERR1817167	Fayoumi	Female	23	Trachea	Epithelial cell	LaSota	205 microliters 10^7 embryo infectious dose of 50%	2 dpi	[23]
in vitro	LaSota rep 1	NA	NA	NA	10-day-old SPF chicken embryos	Chicken embryo	Fibroblast cell	LaSota	MOI=1	12 hpi	[20]
in vitro	LaSota rep 2	NA	NA	NA	10-day-old SPF chicken embryos	Chicken embryo	Fibroblast cell	LaSota	MOI=1	12 hpi	[20]
in vitro	LaSota rep 3	NA	NA	NA	10-day-old SPF chicken embryos	Chicken embryo	Fibroblast cell	LaSota	MOI=1	12 hpi	[20]
in vitro	Herts/33 rep 1	NA	NA	NA	10-day-old SPF chicken embryos	Chicken embryo	Fibroblast cell	Herts/33	MOI=1	12 hpi	[20]
in vitro	Herts/33 rep 2	NA	NA	NA	10-day-old SPF chicken embryos	Chicken embryo	Fibroblast cell	Herts/33	MOI=1	12 hpi	[20]
in vitro	Herts/33 rep 3	NA	NA	NA	10-day-old SPF chicken embryos	Chicken embryo	Fibroblast cell	Herts/33	MOI=1	12 hpi	[20]

Supplementary Table 2: L/P coverage ratio as upper bound on the contribution of genomic RNA to the reads, for each sample in our datasets.

Samples			P coverage	L coverage	L:P
**Host**	**Virus**	**Replicate**	**(1887:3074)**	**(8381:14995)**	**Ratio**
in vivo Leghorn (susceptible)	LaSota	1	881.202	35.31293	0.04
		2	6016.106	102.4608	0.017
		3	2856.423	38.40847	0.013
in vivo Fayomi (susceptible)		1	694.4924	20.2972	0.029
		2	238.7239	3.715949	0.016
		3	174.6684	3.451534	0.02
in vitro CEF cells	LaSota	1	21446.46	22403.22	1.045
		2	21193.6	22510.9	1.062
		3	22834.04	24100.62	1.055
	Herts/33	1	132665.8	83765.88	0.631
		2	144995.7	98281.21	0.678
		3	128781.9	96594.86	0.75

Supplementary Table 3: p-values of Student's t-test for differences in fraction of P mRNA.

***p*-value**	**Leghorn**	**Fayoumi**	**CEF Herts/33**	**CEF LaSota**
Leghorn	1.0000000	0.0889002	0.0001608	0.0000055
Fayoumi	0.0889002	1.0000000	0.0001028	0.0000048
CEF Herts/33	0.0001608	0.0001028	1.0000000	0.0001686
CEF LaSota	0.0000055	0.0000048	0.0001686	1.0000000

Supplementary Table 4: combined linear model for log10(mRNA fraction) as a function of experiments and length of insertion.

Coefficient	Estimate	Std. Error	t value	Pr(>|t|)
(intercept)	0.0078917	0.1438051	0.0548776	0.9563945
l (slope)	-0.5291164	0.0360768	-14.6664042	0.0000000
Fayoumi	0.0737049	0.2320661	0.3176032	0.7517451
CEF-LaSota	-0.3953569	0.1830215	-2.1601668	0.0342359
CEF-Herts/33	-0.8453087	0.1990163	-4.2474354	0.0000663
l:Fayoumi	-0.0383042	0.0729569	-0.5250249	0.6012488
l:CEF-LaSota	0.0135007	0.0411087	0.3284146	0.7435928
l:CEF-Herts/33	0.0557983	0.0481860	1.1579794	0.2508650
